# Examination of the spatial-temporal evolution and level measurement of industrial green manufacturing development: A case study of the Yangtze River Economic Belt

**DOI:** 10.1371/journal.pone.0324804

**Published:** 2025-10-06

**Authors:** Zihao Zheng, Xinquan Ge, Zongshui Wang

**Affiliations:** 1 School of Economics, Minzu University of China, Beijing, China; 2 School of Economics and Management, Beijing Information Science and Technology University, Beijing, China; Zhejiang University, JAPAN

## Abstract

With the increasingly serious problems of global climate change and resource tension, green manufacturing has become an important direction of industrial development. This paper constructs an evaluation index system for the development level of industrial green manufacturing based on the panel data of 11 provinces in the Yangtze River Economic Belt from 2018 to 2022, and utilizes the Entropy-GRA-TOPSIS method, the natural breakpoint classification method, Theil index, and Moran’s I to explore the development level, spatiotemporal characteristics, regional differences, and spatial correlation of industrial green manufacturing in the Yangtze River Economic Belt, and puts forward the corresponding suggestions. The results show that: the development level of industrial green manufacturing in the Yangtze River Economic Belt shows an upward trend, with an average annual growth rate of 5%, but there are large regional differences, showing a development pattern of “strong in the east and weak in the west”; the regional spatial differences continue to expand, but shrink in 2022, and the overall differences among the three major regions mainly originate from the inter-regional differences, with the central region having the largest differences and contribution rate, and the central region having the largest differences and contribution rate, and the central region having the largest differences and contribution rate. The overall differences among the three regions mainly come from inter-regional differences, with the central region having the largest differences and contribution rate, and the intra-regional differences and contribution rate also showing a fluctuating growth trend; the green development of industry in the Yangtze River Economic Belt shows a significant positive spatial correlation in general, with the HH-type mostly in the eastern provinces and cities, and the LL-type mostly in the central and western provinces and cities.

## 1 Introduction

Against the backdrop of surging global carbon emissions and accelerating climate warming, the creation of a “green economy” has gained international attention. In 2022, the United Nations Environment Programme (UNEP) has designated “One Planet” as the subject for World Environment Day in an effort to advance global economic transformation and strengthen the governance of the globalized green economy [1]. The European Union (EU) initiated a comprehensive strategy for the development of a green economy in 2009, allocating €105 billion to foster this sector prior to 2013. Concurrently, the United States intensified its efforts to establish a legal framework for the green economy, thereby ensuring legal safeguards for its advancement within the nation. The Great Green Technology Blueprint and other policies that support the environmental protection industry have been introduced by the Malaysian government to encourage the growth of a green economy. Amidst these circumstances, there is a growing national agreement to encourage economic restructuring via technological and institutional innovation, curtail the use of high-carbon energy, and expeditiously achieve the goal of advancing environmental conservation and economic development in tandem [2]. China has also clearly listed green manufacturing as a national strategy.

The Yangtze River Economic Belt is a significant economic area in China, and its degree of industrial growth has a direct impact on the nation’s economic power and global competitiveness [3]. The Yangtze River Economic Belt will have more than 60% of its area urbanized by 2019. However, the environmental and ecological destruction that comes along with industrialization and urbanization is becoming more apparent as they expand faster than before [4]. The Ministry of Industry and Information Technology’s (MIIT) General Office published a Notice on Green Manufacturing System in 2016 to implement the policy of green manufacturing [5]. The notice involved the development of pertinent standards and assessment mechanisms. Implementation Programs for Green Manufacturing System Construction have been developed in several regions. The Guiding Opinions on Strengthening the Green Development of Industry in the Yangtze River Economic Belt, released jointly by five departments—among them the Ministry of Industry and Information Technology (MIIT)—call for modifying the industrial structure, encouraging the greening of the traditional manufacturing sector, and lessening the effects of manufacturing development on the ecological environment [6]. The Yangtze River Economic Belt is designated as China’s primary theater of ecological conflict and green development. The Ministry of Industry and Information Technology has also promulgated the “14th Five-Year Plan” for the Green Development of Industry, which emphasizes the need to support the low-carbon and green development of the traditional manufacturing sector [7]. In this regard, green manufacturing serves as a new paradigm for industrial development that strives for environmentally friendly protection of the environment and effective use of resources, offering fresh perspectives for the long-term growth of enterprises in the Yangtze River Economic Zone. Building green manufacturing evaluation index systems, green manufacturing evaluation techniques and the spatial and temporal evolution of the development level of green manufacturing are the primary focus of the current scholarly assessment of the state of industrial green manufacturing development [8].

Research on industrial green manufacturing mainly focuses on the following aspects. First, in constructing the indicator system. Scholars’ research on green manufacturing evaluation indexes has provided a multi-faceted perspective. Continuous enrichment of the evaluation system based on its predecessors. For example, Hermann [9] provided a comprehensive view, and he emphasized the centrality of whole-life-cycle evaluation in green manufacturing, and that every aspect of the process, from product design, production, manufacturing, and consumption, to recycling, should be included in the evaluation system. This view not only provided a theoretical basis for the implementation of green manufacturing but also pointed out the direction for subsequent research. QR He’s study [10] further deepened this concept by proposing a green supplier evaluation system that covers four key dimensions: ability to meet the manufacturer’s needs, relationship, responsibility, commitment, and ability to implement environmental management. Liu [11] examined the green manufacturing system from four perspectives, namely, green factories, green products, green parks, and green supply chains. They provided a comprehensive description of the green manufacturing system from four aspects, and his study pointed out the deficiencies in China’s product life cycle assessment methodology, system boundaries, and life cycle phases, especially the lack of green product evaluation criteria. Bui TD’s study [12] further expanded the scope of the evaluation indicators of green manufacturing, and he constructed a green manufacturing model that contains five aspects and 23 criteria, arguing that manufacturers should focus on clean technology, responsibility, commitment, and implementation of environmental management. It was argued that manufacturers should focus on clean technologies, green processes, life cycle management, waste recycling, and textile waste generation and management. These findings provide concrete operational guidelines for green manufacturing practices, as well as a valuable reference for policymakers. Second, in terms of measurement methods. A series of research works by scholars have provided a variety of analytical tools and methods for evaluating the level of development of green manufacturing. Measurement methods include entropy weighting, analytic hierarchy process, fuzzy analysis, DEA model, SFA model, and other methods. For example, Sarkis [13] innovatively applied a combination of network analysis and data envelopment analysis to analyze and evaluate the choice of materials in green product design, which provided quantitative decision support for green design. Sezen and Cankaya [14] used regression analysis to study the performance of green manufacturing in the economic and social development of enterprises, the study included more than fifty companies in the manufacturing industry in Turkey, and the results of the study showed that green manufacturing has a positive impact on the sustainability of the manufacturing industry, thus providing a strong evidence for the sustainable development of the manufacturing industry. Oh D H et al [15] used the ML index to measure green productivity in 26 countries and found that initially green growth is mainly influenced by technical efficiency, and the impact of technological advancement on green productivity becomes more significant over time. Salem et al. [16] took the enterprise as a whole, adopted the geometric mean method (GMM) as a common assessment technique for greening assessment across industries, and used data envelopment analysis (DEA) to assess the intra-industry layer’s greening level, and designed a toolkit for evaluating the greening level of enterprises, this comprehensive evaluation method provides a new perspective on greening assessment across industries and intra-industry layers. Guo et al. [17] innovatively introduced the SFA model into the evaluation framework of urban sustainable development, which more accurately describes the sustainable development of cities and expands the theoretical and practical research related to urban sustainable development. Application to the study of green total factor productivity in Chinese cities. The Analytical Hierarchy Process (AHP) and the Oscillating Value Process (OVP) were combined to create the AHP-OVP evaluation model [18]. The results demonstrate that the AHP-OVP evaluation model can effectively evaluate the capacity for green innovation of manufacturing enterprises and offers strong decision-making support for the green transformation of enterprises. Third, in terms of spatio-temporal evolution, many scholars further analyze it mainly from two perspectives: spatio-temporal evolution characteristics and regional differences. On the one hand, in terms of spatial, and temporal evolution characteristics, some scholars mainly use methods such as kernel density estimation, Moran index and Markov chain. For example, Jin et al. [19] combined the SFA model with the Moran Index to analyze the urbanization efficiency of 110 cities within the Yangtze River Economic Belt from 2005 to 2014, highlighting the spatial and temporal evolution and spatial morphology characteristics of these agglomerations, and found that the urbanization efficiency has a positive spatial correlation with the annual increment in the level of the urban agglomeration. Jin et al. [20] used the kernel density method and spatial autocorrelation method to discuss the spatial and temporal carbon emission efficiency characteristics of 105 cities in the Yangtze River Economic Belt and found that the cities in the upstream, midstream, and downstream of the Yangtze River Economic Belt were concentrated in the lower efficiency group, low-efficiency group, and high-efficiency group, respectively. In order to reveal the evolution pattern and internal relationship of grey water footprint efficiency in both temporal and spatial dimensions, XU et al. [21] employed kernel density curves, a Markov probability transformation matrix, and the Moran index to examine the spatial and temporal evolution characteristics of grey water footprint efficiency in 11 provinces and cities within the Yangtze River Economic Belt. On the other hand, in terms of regional differences, scholars mainly use the Theil index, Gini coefficient, and other methods to verify that there are significant differences in different regions. For example, Wang et al. [22] combined the entropy TOPSIS model and the Theil index to analyze the differences between the industrial green development of China’s three major urban agglomerations and found that the differences in green development between Beijing-Tianjin-Hebei and the Yangtze River Delta were the smallest. The industrial green development of Beijing-Tianjin-Hebei cities is more balanced. Yang et al. [23] used the Dagum spatial Gini coefficient to assess the green resilience of 286 prefectural-level cities in China from 2012 to 2021 and found that the gap in green resilience between the central and western regions of China is consistently the largest, followed by the east and west, and the central and east regions are the smallest.

To summarize, the existing literature on industrial green manufacturing-related research has made many achievements, which has laid an important theoretical and methodological foundation for industrial green manufacturing research, but there is also room for further deepening and expansion at the same time. First of all, this study has sorted out the evaluation objects of green manufacturing, and the evaluation studies mainly focus on the national and enterprise levels, less research has been done expressly on the features of the chronological and spatial evolution of the growth of industrial green manufacturing in a particular province or area. Second, from the perspective of the measurement model, prior research has mostly used a single evaluation framework or model, such as entropy weighting, hierarchical analysis, fuzzy analysis, data envelopment analysis, etc., to assess the degree of industrial green manufacturing development [24]. Future evaluation research, however, will likely combine several approaches in various assessment frameworks, which not only allows for a deeper examination of complex systems but also adds a variety of viewpoints to the study [25]. Furthermore, the current analysis of regional disparities in the degree of industrial green manufacturing development within the Yangtze River Economic Belt remains superficial, making it challenging to discern the underlying regional correlations and the specific contributions to the disparities in the level of industrial green manufacturing development. Finally, in recent years, the promotion of green manufacturing has been gradually accelerated and diffused, and the existing research on green manufacturing development indicators can not keep pace with the times, and lack of timeliness and comprehensiveness.

**Fig 1:**
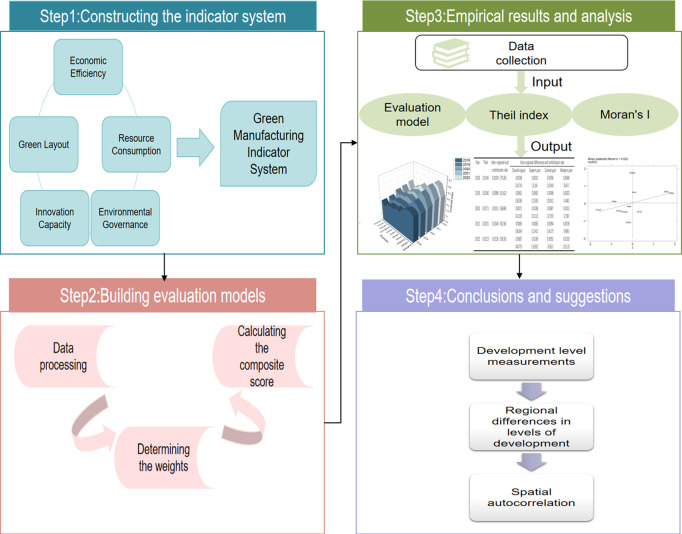
Research framework for spatial-temporal evolution and level measurement of industrial green manufacturing development.

The contribution of this research is mainly reflected in the following aspects. Firstly, the innovation of research perspective, this study selects the Yangtze River Economic Belt as the research area, aiming at quantitatively assessing the development level of industrial green manufacturing in the region, thus providing a new perspective and data support for the study of industrial green manufacturing. The time period from 2018 to 2022 was chosen as the study period in order to capture the most recent trends and developments in the growth of industrial green manufacturing in the Yangtze River Economic Belt while accounting for the completeness and availability of the data. This is because some core statistics are not fully publicized after 2022, and some core statistics are incomplete before 2018. Secondly, innovation in research methodology, The entropy weight approach is used in this study to establish the weights and build the Entropy-GRA-TOPSIS model, which combines the benefits of TOPSIS and grey correlation analysis (GRA). This not only offers an alternative viewpoint to the study but also makes it easier to examine complicated systems in depth. Additionally, the Thiel index and Moran’s I are used to study the regional and intra-regional variations in the level of industrial green manufacturing development in the Yangtze River Economic Belt, and the spatial spillover effects are further investigated. Thirdly, the innovation of evaluation indexes, in order to make the evaluation indexes more current and comprehensive, this paper adds new indicators that are up-to-date with the times, such as Green Supply Chain Management Enterprise and Land use output, etc., and establishes a more comprehensive green manufacturing evaluation index, as shown in Fig 1.

This paper attempts to address the following questions: what are the patterns of the temporal and spatial evolution of industrial green manufacturing development in the Yangtze River Economic Belt at the province spatial scale? Inter- and intra-regional differences and their spatial agglomeration characteristics in the Yangtze River Economic Belt. By answering the above questions, on the one hand, the connotation of green manufacturing can be further enriched and expanded, which is conducive to improving the research system of industrial green manufacturing and has certain theoretical value. On the other hand, it can provide useful reference and references for the better coordination of regional industrial green manufacturing complementary win-win situation and coordinated development.

## 2 Construction of indicator system

The ideal mode and direction of China’s manufacturing industry’s future development are made clear by green manufacturing, and determining whether it is feasible to create a reasonable and scientific indicator system is essential to assessing the state of green manufacturing development reasonably and scientifically [26]. To comprehensively and systematically evaluate the development level of industrial green manufacturing, this paper, on the premise of following the principles of orientation, fairness, scientificity, feasibility, and systematicity of constructing the index system, and based on the research of relevant experts and scholars, and regarding the “14th Five-Year Plan for Green Development of Industry” issued by the Ministry of Industry and Information Technology, an appropriate and scientific index technique for assessing the degree of industrial green manufacturing was constructed by selecting 18 indicators in five dimensions, namely, Economic efficiency, Resource consumption, Environmental governance, Innovation capacity, and Green layout [27]. As shown in Table 1.

**Table 1:** Evaluation system for industrial green manufacturing development level.

Target layer	Criterion layer	Label	Meaning	Weight	Direction	Reference
Economic benefit	Growth rate of IVA(%)	A1	IVA Variance/Previous Period IVA	0.0130	+	[3,28]
	IVA in GDP(%)	A2	IVA/GDP	0.0287	+	
	Industrial operating margin(%)	A3	Total profit/Revenues	0.0860	+	
	Land use output(%)	A4	IVA/Amount of industrial land	0.0545	+	
Resource consumption	Energy consumption (10000 TCE)	B1	Industrial energy consumption/IVA	0.0180	_	[29,30]
	Water consumption(10,000 cubic meters)	B2	Industrial water consumption/IVA	0.0332	_	
	Electricity consumption(100 million kWh)	B3	Industrial electricity consumption/IVA	0.0177	_	
Environmental governance	Amount of investment in pollution control(10000 Yuan)	C1	Investment in industrial pollution control completed/IVA	0.0641	+	[31,32]
	SO2 emissions(ton)	C2	Industrial SO2 emissions/IVA	0.0076	_	
	Wastewater emissions(ton)	C3	Industrial wastewater emissions/IVA	0.0164	_	
	Solid waste utilization rate(%)	C4	Industrial solid waste utilization/Industrial solid waste generation	0.0482	+	
Innovation capacity	R&D intensity of expenditure(%)	D1	R&D funding/GDP	0.0549	+	[11,33]
	R&D personnel as a proportion of employees(%)	D2	Number of R&D personnel in industrial enterprises/Number of persons employed in industry	0.0421	+	
	Number of active patents(piece)	D3	Active patents are those whose inventions deal with green technology, including pollution avoidance, energy efficiency, resource conservation, and control.	0.1284	+	
Green layout	Number of Green factory(unit)	E1	Low-carbon energy, clean production, resource efficiency, safe raw material use, and intensive land utilization are the goals of Green factory	0.0433	+	[34,35]
	Number of Green Industrial Park (unit)	E2	Green industrial park refers to a park that combines intense and economical land use with cascading energy and water recycling, trash exchange and usage, and interchange	0.0482	+	
	Number of Green Design Products(piece)	E3	Green design products use less energy and resources, are safe or less hazardous to human health and the environment, and maintain a high standard of quality throughout their whole life cycle	0.1308	+	
	Number of Green Supply Chain Management Enterprise(unit)	E4	Green supply chain management enterprises are those that actively incorporate the concepts of environmental protection, resource conservation, and health and safety in their supply chain management	0.1648	+	

## 3 Data and methods

### 3.1 Study area

The Yangtze River Economic Belt covers 205,230 km^2^, approximately 21.4% of the country’s total area, and is made up of 11 provinces and municipalities: Shanghai, Jiangsu, Zhejiang, Anhui, Jiangxi, Hubei, Hunan, Chongqing, Sichuan, Guizhou, and Yunnan [36]. This region is home to over 40% of the country’s population and contributes more than 40% to the national gross domestic product (GDP). According to the regional division of the Outline of the Strategic Plan for the Development of the Yangtze River Economic Belt, the Yangtze River Economic Belt is divided into three parts, with the eastern part containing Shanghai, Jiangsu, and Zhejiang, the central part containing Anhui, Jiangxi, Hunan and Hubei, and the western part containing Sichuan, Chongqing, Yunnan, and Guizhou(2). As shown in Table 2.

**Table 2:** Regional division of the Yangtze River Economic Belt.

Region	Provinces
Eastern part	Shanghai, Jiangsu, Zhejiang
Central part	Anhui, Jiangxi, Hunan, Hubei,
Western part	Sichuan, Chongqing, Yunnan, Guizhou

### 3.2 Data sources

Eleven cities and provinces in the Yangtze River Economic Belt have been chosen as the study’s subjects., and the regional statistical yearbook lacks data for 2023 and beyond, so the research period is 2018–2022, and the relevant data come from the provincial and municipal statistical yearbooks, ecological environment bulletins and China Statistical Yearbook, China Energy Statistical Yearbook, China Environmental Yearbook, etc. This study ensures the reliability and accuracy of the research data by utilizing data sources of equivalent statistical caliber. Missing data were estimated using linear interpolation [37].

### 3.3 Mothods

#### 3.3.1 Entropy-GRA-TOPSIS method.

The entropy weight method uses information entropy to determine the weight, which evaluates the importance of indicators by calculating the information entropy of the indicators and then determines the weights [38], which makes the determination of the weights more objective and avoids the bias brought by the human factor, however. The entropy weight method treats distinct indicators as independent entities, disregarding their intercorrelations, which may lead to an unjust allocation of weights.

TOPSIS, a multi-attribute decision-making analysis method, assesses a system’s performance by computing the weighted Euclidean distances to both the positive and negative ideal solutions. This approach is noted for its simplicity in concept and computation, and it enjoys widespread application across diverse research domains, however, the method can only reflect the relative positional relationship of the data curves, and can not embody the system’s dynamic changes.

The grey relation analysis method for the data requirements is relatively low, the workload is relatively small, and can largely reduce the loss due to information asymmetry, but the method requires the need for the optimal value of the indicators to determine the current, too subjective, and some of the indicators optimal value is difficult to determine.

The entropy weight approach is applied to give weights to the indexes according to the properties of the object to be evaluated. To overcome the entropy weight method’s neglect of the indexes’ meaning, the TOPSIS method is combined with the grey relation analysis method. This not only compensates for the shortcomings of both methods but also combines their advantages to create a more thorough evaluation of the object to be evaluated.

Step 1 Data standardization

Positive indicators


$$\begin{array}{c} {}*20cX_{ij}=\frac{x_{ij}-\min\left\{x_{1j},\ldots ,x_{nj}\right\}}{max\left\{x_{1j},\ldots ,x_{nj}\right\}-min\left\{x_{1j},\ldots ,x_{nj}\right\}} \end{array}$$ (1)

Negative indicators


$$\begin{array}{c} {}*20cX_{ij}=\frac{max\left\{x_{1j},\ldots ,x_{nj}\right\}-x_{ij}}{max\left\{x_{1j},\ldots ,x_{nj}\right\}-min\left\{x_{1j},\ldots ,x_{nj}\right\}} \end{array}$$ (2)

In the formula, i is the subject of the evaluation and j is the evaluation indicator; $X_{ij}$ is the original value of the jth indicator of evaluation object i; n is the number of evaluation objects; $max\left\{x_{1j},\ldots ,x_{nj}\right\}$ and $min\left\{x_{1j},\ldots ,x_{nj}\right\}$ are the maximum and minimum values of the jth indicator data, respectively.

Step 2 Entropy weights to determine weights.


$$\begin{array}{c} {}*20c\omega_{j}=\frac{1-e_{j}}{\sum {\mathrm{\backslash nolimits}}_{i=1}^{m}\left(1-e_{j}\right)} \end{array}$$ (3)

$$\begin{array}{c} {}*20ce_{j}=-\frac{1}{\ln\left(n\right)}\sum_{i=1}^{n}p_{ij}ln\left(p_{ij}\right) \end{array}$$ (4)

$$\begin{array}{c} {}*20cp_{ij}=\frac{X_{ij}}{\sum {\mathrm{\backslash nolimits}}_{i=1}^{n}X_{ij}} \end{array}$$ (5)

In the formula, $e_{j}$ is the information entropy of the jth indicator; $p_{ij}$ is the weight of the jth indicator of evaluation object i; m is the number of indicators; n is the number of evaluation objects; $\omega_{j}$ is the indicator weight.

Step 3 Construct a weighted decision matrix and determine the ideal solution.


$$\begin{array}{c} {}*20cr_{ij}=w_{j}\times x_{ij},r_{ij}\in R \end{array}$$ (6)

positive ideal solution


$$\begin{array}{c} {}*20cR_{j}^{+}=\left\{r_{1}^{+},r_{2}^{+},r_{3}^{+},\ldots ,r_{n}^{+}\right\} \end{array}$$ (7)

negative ideal solution


$$\begin{array}{c} {}*20cR_{j}^{-}=\left\{r_{1}^{-},r_{2}^{-},r_{3}^{-},\ldots ,r_{n}^{-}\right\} \end{array}$$ (8)

In equations (7) to (8), $R_{j}^{+}$ and $R_{j}^{-}$ are the set of maximum and minimum values of each indicator; $r_{ij}$ is the weighted decision matrix.

Step 4 Between the evaluation object and the positive (negative) ideal solution, compute the matrix of gray correlation coefficients.


$$\begin{array}{c} {}*20c\rho_{ij}^{+}=\frac{\underset{i}{min}\underset{j}{min}\left|r_{j}^{+}-r_{ij}\right|+\varepsilon \times \underset{i}{max}\underset{j}{max}\left|r_{j}^{+}-r_{ij}\right|}{\left|r_{j}^{+}-r_{ij}\right|+\varepsilon \times \underset{i}{max}\underset{j}{max}\left|r_{j}^{+}-r_{ij}\right|} \end{array}$$ (9)

$$\begin{array}{c} {}*20c\rho_{ij}^{-}=\frac{\underset{i}{min}\underset{j}{min}\left|r_{j}^{-}-r_{ij}\right|+\varepsilon \times \underset{i}{max}\underset{j}{max}\left|r_{j}^{-}-r_{ij}\right|}{\left|r_{j}^{-}-r_{ij}\right|+\varepsilon \times \underset{i}{max}\underset{j}{max}\left|r_{j}^{-}-r_{ij}\right|} \end{array}$$ (10)

In equations (9) to (10), $\rho_{ij}^{+}$ and $\rho_{ij}^{-}$ are the grey correlation coefficients between the jth indicator of the evaluation object i and the positive (negative) ideal solution;$\;r_{j}^{+}$ and $r_{j}^{-}$ are the maximum and minimum values of the jth indicator; ε ∈ (0,1), is the discrimination coefficient, which takes the value of 0.5 in this study.

The grey relational grade between the evaluation object i and the positive (negative) ideal solution can be determined from this analysis:

Positive ideal solution grey correlation


$$\begin{array}{c} {}*20cr_{i}^{+}=\frac{1}{m}\times \sum_{j=1}^{m}\rho_{ij}^{+} \end{array}$$ (11)

Negative ideal solution grey correlation


$$\begin{array}{c} {}*20cr_{i}^{-}=\frac{1}{m}\times \sum_{j=1}^{m}\rho_{ij}^{-} \end{array}$$ (12)

In equations (11) to (12), m is the number of indicators, $\rho_{ij}^{+}$ and $\rho_{ij}^{-}$ are the gray correlation coefficients between the evaluation object and the positive (negative) ideal solution calculated in step 4; $r_{i}^{+}$ and $r_{i}^{-}$ are the gray correlation degrees of the positive (negative) ideal solution, respectively.

Step 5 Determine the assessment object’s grey correlation closeness.


$$\begin{array}{c} {}*20c\eta_{i}=\frac{r_{i}^{+}}{r_{i}^{+}+r_{i}^{-}},i=1,2,\ldots ,n \end{array}$$ (13)

In equations (13), $\eta_{i}$ denotes the gray correlation posting progress of evaluation object i. The assessment of the development level of regional industrial green manufacturing can be achieved according to the grey correlation index, and the larger. $\eta_{i}$ Indicates that the assessment results are better.

#### 3.3.2 Theil index and decomposition.

Theil index as a kind of relative difference index was first put forward by Theil in 1967 based on the concept of entropy in the theory of information, and its basic purpose is to compare the contribution and influence of different sub-districts of the region on the overall differences of the whole region by decomposing the overall differences of a certain region into the differences of the two components of the differences of the indicators of the differences of the group and the differences of the groups, and this method of gauging the extent of regional disparity [39]. This research utilizes the Theil index to evaluate the disparities in industrial green manufacturing development across various regions, including both intra-regional and inter-regional differences, and to ascertain the corresponding contribution rates [40]. This is achieved by analyzing the extent of variation both within and between regions.


$$\begin{array}{c} {}*20cT=\frac{1}{k}\sum_{q=1}^{k}\left(\begin{array}{c} {}*20c\frac{S_{q}}{\bar{S}}\times ln\frac{S_{q}}{\bar{S}} \end{array}\right) \end{array}$$ (14)

In the formula, T indicates the overall difference of regional industrial green development of the Terrell index, the size of which is in [0, 1], the smaller Theil index, indicating that the overall difference of industrial green manufacturing development is smaller, and vice versa, indicating that the overall difference is larger [41]. q indicates the province, k indicates the number of provinces, $S_{q}$ indicates the level of development of industrial green manufacturing in the province q, and $\bar{S}$ indicates the average of the regional level of development of industrial green manufacturing.


$$\begin{array}{c} {}*20cT_{p}=\frac{1}{k_{p}}\sum_{q=1}^{k_{p}}\left(\frac{S_{pq}}{{\bar{S}}_{p}}\times ln\frac{S_{pq}}{{\bar{S}}_{p}}\right) \end{array}$$ (15)

In the formula, p denotes a region; $T_{p}$ denotes the Theil index of overall variation in region p, Where $T_{p}$ denotes the overall difference Theil index of region p, $k_{p}$ denotes the number of provinces in region p, $S_{pq}$ denotes the level of industrial green manufacturing development in province q of region p, and ${\bar{S}}_{p}$ denotes the mean value of industrial green manufacturing development level in region p.


$$\begin{array}{c} {}*20cT=T_{w}+T_{b}=\sum_{p=1}^{3}\left(\frac{k_{p}}{k}\times \frac{{\overset{―}{S}}_{p}}{\overset{―}{S}}\times T_{p}\right)+\sum_{p=1}^{3}\left(\frac{k_{p}}{k}\times \frac{{\overset{―}{S}}_{p}}{\overset{―}{S}}\times ln\frac{{\overset{―}{S}}_{p}}{\overset{―}{S}}\right) \end{array}$$ (16)

In the formula, the Theil index of the overall difference in industrial green manufacturing development is further decomposed into the within-region difference Theil index. $T_{w}$ and the between-region difference Theil index $T_{b}$ In addition, T_w_/T and T_b_/T are defined as the contribution of the within-region difference and the between-region difference to the overall difference, respectively, and (S_p_/S)*(T_p_/T) is the contribution of the regions to the overall difference within the region, and S_p_ denotes the sum of industrial green manufacturing development levels of the provinces in region p, and S denotes the sum of the overall regional industrial green manufacturing development levels. Provinces within regions p, and S denotes the sum of industrial green manufacturing development levels in the overall region.

#### 3.3.3 Moran’s I.

According to the first law of geography, everything has a certain correlation, and the correlation increases with distance [42]. When examining spatial relationships, one might use Moran’s I, a correlation coefficient whose value range is [−1, 1]. An exceedance of 0 in the values signifies a positive spatial autocorrelation in the dataset, where greater values suggest more pronounced correlations. Moran’s I includes two components: Local Moran’s I and Global Moran’s I. Global Moran’s I is used to determine whether a spatial correlation exists overall; if it does, the spatial correlation will be more evident; Local and Global Moran’s I are included in Moran’s I. Global Moran’s I is used to assess whether there is geographical connection overall; if it demonstrates importance, it can be subjected to additional analysis Local Moran’s I[43]. This study employs Moran’s I to assess the spatial autocorrelation of the regional industrial green manufacturing development levels. Global Moran’s I reveals the overall degree of correlation of regional spatial attributes, and its specific formula is as follows:


$$\begin{array}{c} {}*20cI=\frac{n\sum {\mathrm{\backslash nolimits}}_{i=1}^{n}\sum {\mathrm{\backslash nolimits}}_{j=1}^{n}w_{ij}\left(y_{i}-\bar{y}\right)\left(y_{j}-\bar{y}\right)}{\left(\sum {\mathrm{\backslash nolimits}}_{i=1}^{n}\sum {\mathrm{\backslash nolimits}}_{j=1}^{n}w_{ij}\right)\sum {\mathrm{\backslash nolimits}}_{i=1}^{n}{\left(y_{i}-\bar{y}\right)}^{2}} \end{array}$$ (17)

The following formula, obtained from the Local Moran’s I, indicates the spatial correlation patterns among various regions:


$$\begin{array}{c} {}*20cI_{i}=\frac{y_{i}-\bar{y}}{\frac{1}{n}\sum {\left(y_{i}-\bar{y}\right)}^{2}}\sum_{j\neq i}^{n}w_{ij}\left(y_{j}-\bar{y}\right) \end{array}$$ (18)

In both equations, n is the total number of regions, i and j denote the object of observation; $y_{i}$ and $y_{j}$ denote the attribute values of observation i, and observation j respectively; $\bar{y}\;$ is the average value of the industrial green manufacturing development index of all regions, and $w_{ij}$ is the spatial weight matrix based on the proximity of provinces.


$$\begin{array}{c} {}*20cw_{ij}=\{\begin{array}{cc} {}*20l1 & \left(Areas\;i\;and\;j\;are\;spatially\;adjacent\right)\\ 0 & \left(Areas\;i\;and\;j\;are\;not\;spatially\;contiguous\right) \end{array} \end{array}$$ (19)

## 4. Results

### 4.1 Analysis of development level measurements

#### 4.1.1 Time evolution analysis.

The industrial green manufacturing development level of 11 provinces and cities in the Yangtze River Economic Belt is indexed using Entropy-GRA-TOPSIS, and the results are ranked according to the index for each year between 2018 and 2022. The evaluation index system for industrial green manufacturing development levels is constructed employing the entropy weight method to determine the weights of the indicators, as shown in Table 3.

**Table 3:** Comprehensive Evaluation Score and Ranking of Green Manufacturing Development Level of Industries in the Yangtze River Economic Belt.

Provinces	Value of Comprehensive Evaluation						
	2018	2019	2020	2021	2022	Mean	Ranking
Shanghai	0.4318	0.4054	0.4268	0.4752	0.4583	0.4395	4
Jiangsu	0.4776	0.4903	0.5601	0.5935	0.6176	0.5478	2
Zhejiang	0.4735	0.5318	0.6091	0.6505	0.6432	0.5816	1
Anhui	0.4372	0.4648	0.5042	0.5341	0.4660	0.4813	3
Jiangxi	0.3510	0.3341	0.3573	0.3711	0.3715	0.3570	10
Hubei	0.3716	0.3442	0.3837	0.4068	0.4422	0.3897	6
Hunan	0.3307	0.3524	0.4113	0.4364	0.4915	0.4045	5
Chongqing	0.3377	0.3540	0.3785	0.4085	0.4207	0.3799	8
Sichuan	0.3245	0.3515	0.3827	0.3782	0.3946	0.3663	9
Yunnan	0.2714	0.2997	0.3484	0.3014	0.2973	0.3036	11
Guizhou	0.3414	0.3484	0.4017	0.4169	0.4397	0.3896	7
Mean	0.3771	0.3888	0.4331	0.4521	0.4584	0.4219	

**Fig 2:**
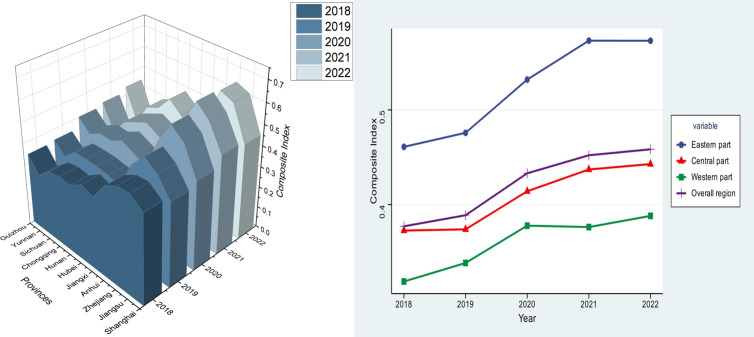
Trends in the development level of industrial green manufacturing in the Yangtze River Economic Belt.

As shown in Fig 2, Significant regional disparities are observed in the levels of industrial green manufacturing development across cities within the Yangtze River Economic Belt. Specifically, Zhejiang has the highest level in 2021 with an indicator value of 0.6505, while Yunnan has the lowest level in 2018 with an indicator value of 0.2714. From a regional perspective, the trend of the development level of industrial green manufacturing during the study period was the same in the eastern, central, and western parts of the Yangtze River Economic Belt, with the whole showing a steady rise. Of these, the comprehensive score of industrial green manufacturing in the eastern region is higher than the level of the Yangtze River Economic Belt as well as in the central and western regions, rising from 0.4610 in 2018 to 0.5730 in 2022. Overall, the development level of industrial green manufacturing in the Yangtze River Economic Belt exhibits an upward trend with an average annual growth of 5%. The industrial green manufacturing score in both the western and central regions has been growing at a slower rate than the average level of the Yangtze River Economic Belt for some time. In 2018, the central region’s score increased from 0.3726 to 0.4428, while the western region’s score increased from 0.3188 to 0.3881. There is a significant disparity in the industrial green manufacturing development levels across the region, indicating a “strong in the east and weak in the west” pattern of development. As the major cities in the Yangtze River Economic Belt, Zhejiang, Jiangsu, Hunan, and Anhui have a strong industrial base, a reasonable industrial structure, and a superior geographical location when viewed from the perspective of the Yangtze River Economic Belt’s most recent level of industrial green manufacturing development in 2022. In addition, the four provinces and cities have a large number of colleges and universities with a focus on industry as well as scientific research institutes, which offer strong intellectual support for green manufacturing. Shanghai is also located in the Yangtze River Economic Belt, and while being radiated and driven, it optimizes the trade structure, promotes the trade of high-quality, high-efficiency, and high-value-added green products, strengthens the environmental sustainability of overseas cooperation projects, and strengthens the cooperation between Shanghai and the “Belt and Road” countries in the fields of green infrastructure, green energy, green finance, energy saving and environmental protection, etc. Therefore, the development level of Shanghai’s industrial green manufacturing is also at the forefront. Among the 11 provinces and municipalities in the Yangtze River Economic Belt, Yunnan, Jiangxi, Sichuan, and Chongqing rank lowest in terms of industrial green manufacturing development. From the perspective of the whole research cycle, Yunnan’s industrial green manufacturing development level has lagged somewhat. This is primarily because Yunnan Province’s overall economic development is relatively low, a significant portion of traditional industries exist, and the industrial base is weak, making it more difficult to optimize and upgrade the industrial structure. Additionally, Yunnan’s plateau location and relatively complex topography present additional challenges for the province’s industrial layout and logistics and transportation, which in turn hinder the growth of the industrial green manufacturing sector. Jiangxi Province, a significant province in central China, has been actively promoting industrial green manufacturing in recent years. However, the province still lags behind other developed regions in terms of development due to its traditional industrial structure, which primarily consists of heavy and resource-based industries that frequently require a lot of energy and resources during production The production processes of these industries result in high energy consumption and environmental pollution [44]; additionally, the green manufacturing service system is not yet flawless, necessitating the strengthening of professional talent and technical support. Taken together, these factors account for Jiangxi Province’s relative lag in the development of industrial green manufacturing. Sichuan Province and Chongqing Municipality, the industrial hub of the southwest, clearly lag behind the eastern coastline region in terms of green manufacturing technology research, development, and implementation. The development of industrial green manufacturing in these two provinces and municipalities has also been relatively low due to a lack of endogenous motivation for transformation and upgrading, a lack of strong enterprise awareness, and a relatively low level of attention and willingness to invest in green manufacturing.

#### 4.1.2 Spatial distribution characteristics.

In the Yangtze River Economic Belt provinces, the industrial green manufacturing development level has evolved over the last five years. The characteristics of this evolution were examined by visualizing the development-level data in terms of spatial distribution. The Yangtze River Economic Belt’s industrial green manufacturing development level is split into five categories using the ArcGIS natural breakpoint approach: low, lower, medium, higher, and high.

From 2018 to 2022, the Yangtze River Economic Belt’s industry’s total green manufacturing development index indicates a steady development trend. This study identifies two significant characteristics regarding industrial green manufacturing development within the Yangtze River Economic Belt: firstly, pronounced regional disparities exist between areas with high and low development levels. High-level development is predominantly found in central and eastern regions, including Jiangsu, Zhejiang, Shanghai, and Anhui, whereas low-level development is mainly observed in western regions, such as Yunnan. The spatial distribution pattern of the declining level of development in the region runs from east to west; second, with the advance of time, the high level of industrial green manufacturing development in the Yangtze River Economic Belt as well as the higher level of the region gradually expanded from east to west to Guizhou and Chongqing, and the higher level and high level of provinces increased over time.

### 4.2 Analysis of regional differences in levels of development

To investigate regional variations in the state of industrial green manufacturing development, the 11 provinces and cities within the Yangtze River Economic Belt were categorized into three areas: eastern, central, and western, and the Theil index was calculated for each year and further decomposed into inter-domain and intra-domain differences, and their respective contribution rates were calculated, with the results shown in Table 4.

**Table 4:** Theil index and contribution rate of the development level of industrial green manufacturing in the Yangtze River Economic Belt.

Year	Theil	Inter-regional and contribution rate	Intra-regional differences and contribution rate			
			Overall region	Eastern part	Central part	Western part
2018	0.0145	0.0109(75.26)	0.0036 (24.74)	0.0010 (2.34)	0.0056 (13.94)	0.0040 (8.47)
2019	0.0160	0.0098(61.62)	0.0061 (38.38)	0.0062 (13.00)	0.0096 (20.91)	0.0023 (4.48)
2020	0.0171	0.0101(58.88)	0.0071 (41.18)	0.0108 (21.12)	0.0087 (17.65)	0.0013 (2.39)
2021	0.0231	0.0146(63.36)	0.0085 (36.64)	0.0083 (12.41)	0.0094 (14.27)	0.0076 (9.99)
2022	0.0213	0.0126(59.30)	0.0087 (40.70)	0.0106 (16.92)	0.0052 (8.63)	0.0105 (15.13)

Regarding the aggregate disparities, the trajectory of the Theil index for industrial green manufacturing development within the Yangtze River Economic Belt from 2018 to 2022 exhibits a pattern of initial increase followed by a subsequent decline, from 0.0145 in 2018 to 0.0213 in 2021, and then decreases to 0.0213 in 2022. The analysis reveals that spatial disparities in industrial green manufacturing development within the Yangtze River Economic Belt increased during the study period, with a subsequent partial reduction observed in 2022. This is because different regions have developed at different rates. In an effort to safeguard the natural environment of the Yangtze River Basin and enhance the efficiency of industrial resource and energy utilization, the Ministry of Industry and Information Technology, in conjunction with five other departments, issued the “Guiding Opinions on Strengthening the Green Development of Industry in the Yangtze River Economic Belt” in 2018. This policy aims to foster the development of green industries within the economic belt, mitigate the ecological impacts of industrialization, and attain sustainable green growth. The eastern and western regions of the Yangtze River Economic Belt have developed at different rates, with regional differences growing during this time. The “14th Five-Year Plan for Green Development of Industry” was released by the Ministry of Industry and Information Technology in 2022. The document emphasizes the necessity for the traditional manufacturing sector to evolve towards low-carbon and sustainable practices, advocating for the designation of the Yangtze River Economic Belt as China’s foremost region for green development and ecological conservation. The state is also paying increasing attention to the issue of balanced development of green manufacturing in the regional industry, which reduces regional disparities [45]. The decomposition results show that the inter-regional differences’ contribution rate in 2018–2022 is greater than 50%, meaning that the inter-regional differences’ contribution rate is higher than the intra-regional differences’ contribution rate [46]. The findings indicate that inter-regional disparities constitute the predominant source of variation in the development of industrial green manufacturing within the Yangtze River Economic Belt, accounting for as much as 75.26% in 2018. These factors combined to create a certain “Matthew effect” in the level of industrial green manufacturing development. This means that the situation of “the strong are always strong and the weak are always weak” has not significantly improved. The eastern section of the Yangtze River Economic Zone is leading the way in the green development of the manufacturing industry there, with the green total factor productivity of the industry displaying a gradient pattern of decline from east to center to west [36]. Thus, there is an urgent need to find a solution to the problem of synchronized development between regions. Furthermore, the general erratic growth tendency of the intra-regional disparity and contribution rate between 2018 and 2022—which peaked in 2020 at 41.18%—indicates that the issue of intra-regional disparity is growing more and more significant in the modern period.

**Fig 3:**
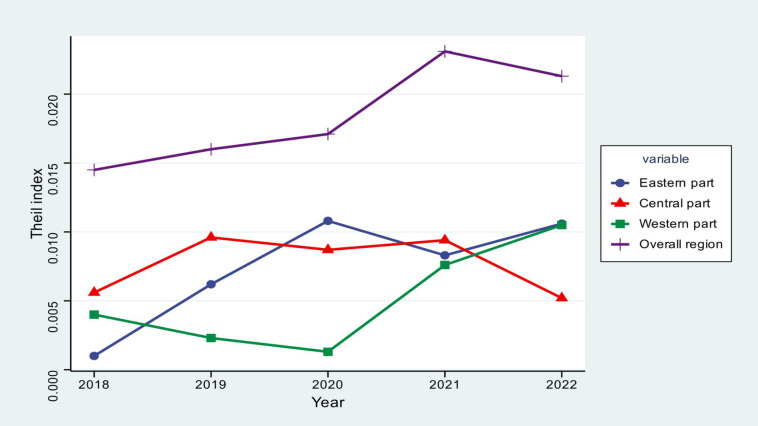
Trends in the Theil index.

As seen in Fig 3, the Theil index for each of the three Yangtze River Economic Belt zones demonstrates distinct patterns throughout time. The decomposition analysis of intra-regional disparities within the Yangtze River Economic Belt reveals that the eastern, central, and western regions exhibited average Theil index values of 0.0074, 0.0077, and 0.0051, respectively, for industrial green manufacturing development levels from 2018 to 2022. The average contribution rate values were 13.16%, 15.08%, and 8.09%, meaning that the central region had the highest intra-regional differences and contribution rates, followed by the eastern region, and comparatively low in the western region. This disparity is attributed to Anhui Province, a pivotal location within the central region of the Yangtze River Economic Belt, which outperforms other regional provinces and cities in fostering a green manufacturing system, implementing low-carbon and environmentally friendly technologies, and advancing the modernization and optimization of the industrial structure. With Anhui serving as the core, other provinces’ industries do not benefit from the central region’s development policy’s failure to propagate and encourage the growth of green manufacturing.

### 4.3 Spatial autocorrelation analysis

The research indicates that the growth of industrial green manufacturing in the Yangtze River Economic Belt exhibits a more pronounced spatial correlation; thus, this paper uses Moran’s I to further investigate the spatial correlation. Eq (17) is used to compute global Moran’s I. The results are displayed in Table 5, where Z is a multiple of the standard deviation and P is the likelihood that a particular stochastic process produced the observed spatial pattern.

**Table 5:** Global Moran’s I for Industrial Green Manufacturing Development in the Yangtze River Economic Belt, 2018-2022.

Year	Moran’s I	Z	P
2018	0.782	4.247	0
2019	0.611	3.536	0
2020	0.498	3.056	0.001
2021	0.554	3.3	0
2022	0.322	2.178	0.015

The Moran’s I of the industrial green manufacturing development in the Yangtze River Economic Belt from 2018 to 2022 are all greater than 0 and P is less than 0.1. A distinct positive spatial autocorrelation is evident in the distribution of industrial green manufacturing development levels across the Yangtze River Economic Belt [47]; that is, if a province has better industrial green manufacturing development, then there is at least one other better-developed province nearby; on the contrary, if a certain province’s industrial green manufacturing development is low, then there exists at least one poorly developed province around it [48]. From the dynamic trend of Global Moran’s I, its value shows a weak shrinking trend and reaches the lowest in 2022, indicating that the trend of spatial agglomeration and distribution of industrial green manufacturing development in the Yangtze River Economic Belt has slowed down, and although there is an increase in 2021 compared to 2020, it is still decreasing compared to 2018.

**Fig 4:**
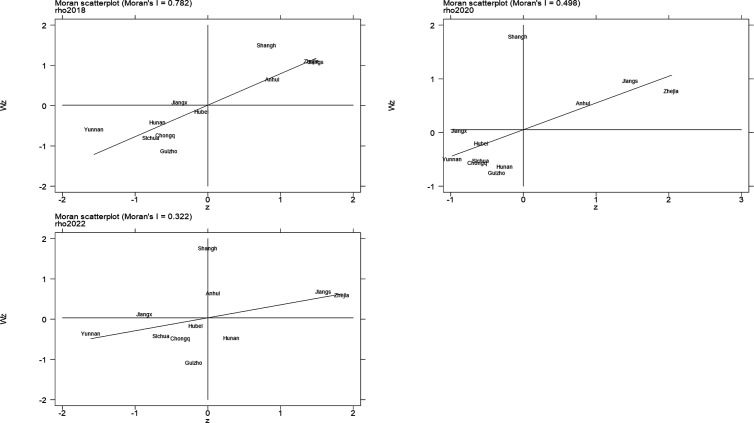
Local Moran scatterplot.

Considering that localities are likely to exhibit characteristics and trends that are different from the overall spatial distribution, this study, with the help of the measurement tool STATA17, used the spatial adjacency matrix to draw the scatter plots of Local Moran’s I for the development of industrial green manufacturing in 11 provincial regions in 2018, 2020, and 2022 (Fig 4), respectively, with the z-value as the x-axis, and the spatial lagged value as the y-axis [49], where the z-value indicates the distance between the comprehensive industrial green manufacturing score of each province and its mean value, the larger the value, the higher the level of industrial green manufacturing in the province [50]; the spatial lag value reflects the spatial correlation of each province, the degree of industrial green manufacturing in the province and its bordering provinces is closer the larger the value [51].

From Fig 4, in the scatterplot of Local Moran’s I of the three representative years, the sample points are mainly concentrated in the first and third quadrants, and the points located in the first quadrant (HH-type) are mainly in the eastern provinces, and cities, indicating that the level of industrial green manufacturing in this part of the region is high and the spatial correlation is also high, such as Zhejiang, Jiangsu, and Shanghai, etc., while the points located in the third quadrant (LL-type) are mainly in the central and western provinces and cities, indicating that the level of industrial green manufacturing in this part of the region is low and the spatial correlation is also high [52]. The points located in the third quadrant (LL-type) are mainly in central and western provinces and municipalities, indicating that the level of industrial green manufacturing in this part of the region is low, and the spatial correlation is also low, such as Chongqing, Sichuan, Yunnan and Guizhou, etc., indicating that China’s “strong in the east and weak in the west” pattern of distribution of the development of industrial green manufacturing, and that the number of provinces and municipalities located in the low-level zone (LL-type) is higher in the three representative years, and the tendency of low low agglomeration is stronger. With the improvement of the development level of some provinces and regions, the number of provinces located in the promotion zone (HH-type) has been improved to a certain extent, and the trend of high and high agglomeration has been strengthened. Overall, there is a certain gap in the development level of industrial green manufacturing among different provinces in the Yangtze River Economic Belt, and the provinces with a higher level of development and those with a lower level of development have formed their agglomeration distribution pattern in space, showing a strong positive correlation, and the positive trend of local spatial agglomeration has been strengthened.

## 5 Discussion

This study reveals the development level, regional differences, and spatial correlations of industrial green manufacturing in the Yangtze River Economic Belt from 2018–2022.

First, the overall development characteristics indicate that the level of industrial green manufacturing in the Yangtze River Economic Belt exhibits an upward trajectory, averaging a 5% annual growth rate. The findings align with Liu’s research [53], which identified an overall upward trajectory for the green development index within the Yangtze River Economic Belt. Additionally, a significant disparity exists in the industrial green manufacturing development levels across the region, adhering to a pattern characterized by robust development in the eastern areas and comparatively weaker development in the western areas. Regional disparities are pronounced in the industrial green manufacturing development levels within the Yangtze River Economic Belt, exhibiting a spatial gradient with a decline in development levels from east to west [54]. This pattern corroborates Liu’s perspective [55], which highlights significant differences in industrial eco-efficiency levels among provinces, and notes that the downstream region’s industrial eco-efficiency average exceeds those of the middle and upper reaches. However, from the perspective of the trend of dynamic change, the region with a high level of industrial green manufacturing development as well as a higher level of development gradually expands and increases from east to west as time advances. The observed phenomenon could be attributed to the increased labor and land costs in the developing eastern coastal region, in contrast to the central and western regions that offer comparatively lower expenses, thereby presenting a more cost-effective environment for manufacturing industries.

Second, regarding regional disparities, the spatial variations in industrial green manufacturing development within the Yangtze River Economic Belt exhibited expansion from 2018 to 2022, with a subsequent contraction in 2022, aligning with the findings of Yang’s study [56]. The overall differences among the three regions are mainly due to interregional differences, and there is a certain “Matthew effect”, which is also supported by Wang’s study [22], which is mainly due to the initial conditions, and the development bases and the degree of resource development in different regions have a significant impact on the development of industrial green manufacturing. The trajectory of intra-regional disparities and their corresponding contribution rates within the Yangtze River Economic Belt exhibits a pattern of fluctuation and growth. The central region experiences the most significant intra-regional disparities and contribution rates, succeeded by the eastern region, whereas the western region records comparatively lower values. Consequently, the issue of intra-regional disparities is gaining increasing prominence. This is consistent with Wang’s view [57]. The prominence of Anhui Province within the central region of the Yangtze River Economic Belt can be attributed to its significant advancements in promoting a green manufacturing system, implementing green low-carbon technological transformations, and optimizing and upgrading the industrial structure, which exceeds those of other provinces and cities in the region. However, the central region’s development policies have not effectively leveraged Anhui Province as a central hub to radiate and stimulate the growth of industrial green manufacturing in other provinces. Finally, when examining the spatial distribution, the industrial green development within the Yangtze River Economic Belt exhibits a notable positive spatial correlation, indicative of spatial spillover effects. Notably, the Moran’s I index value demonstrates a modest decline, reaching its nadir in 2022. This suggests a deceleration in the spatial agglomeration and distribution trends of industrial green manufacturing development in the region, a finding corroborated by Zeng’s research [58]. The Moran scatter plot shows that most of the provinces and cities belong to the HH type or LL type, in which the HH type is mostly in the eastern provinces and cities, such as Zhejiang, Jiangsu, etc., and the central and western provinces and cities are mostly of the LL type, which is similar to Bai’s study [59], and the results show that China’s “strong in the east and weak in the west” pattern of the distribution of the development of green manufacturing in the industry, but the number of provinces of the HH type has increased somewhat with the development of time. The Chinese government’s implementation of the “14th Five-Year Plan for Industrial Green Development” in 2022, which explicitly aimed to foster green and low-carbon advancements in the traditional manufacturing sector and designated the Yangtze River Economic Belt as a pivotal region for China’s ecological and green development priorities, likely contributed to the enhanced development levels of industrial green manufacturing in select provinces within the belt.

It should be pointed out that there are some shortcomings in the research of this paper, for one thing, in the measurement of the development level of industrial green manufacturing, although the selection of evaluation indexes refers to a large number of previous studies, the construction of the index system is not perfect due to the limitations of the availability of data and the existing statistical data. For another, regarding the scope of this study, the research is confined to the five-year timeframe from 2018 to 2022 for 11 provinces and cities within the Yangtze River Economic Belt, due to challenges associated with gathering pertinent information and data. Nevertheless, the study acknowledges that variations in natural resource endowments, socio-economic conditions, and developmental histories across counties and regions introduce inherent limitations. Subsequent research endeavors should focus on enhancing the indicator system and emphasize the examination of industrial green manufacturing development across various temporal and spatial dimensions. Furthermore, investigating the influencing factors of industrial green manufacturing development will contribute to the establishment of a comprehensive research framework, thereby laying a robust theoretical groundwork essential for fostering sustainable urban development.

## 6 Conclusions

This study is based on the basic connotation and development characteristics of industrial green manufacturing, selects 18 indicators from the five dimensions of Economic benefit, Resource consumption, Environmental governance, Innovation capacity, and Green layout, constructs a more timely and comprehensive evaluation index system for the development level of industrial green manufacturing, takes 11 provinces and cities in the Yangtze River Economic Belt as the object of the study, and utilizes Entropy-GRA-TOPSIS method to construct an evaluation model to measure the development level of industrial green manufacturing in the period of 2018–2022, using the natural breakpoint method to draw the spatial evolution map, through Theil index and decomposition to derive the level of industrial green manufacturing development and the source of differences in the regions, and using Moran’s I to analyze the spatial correlation of the industrial green manufacturing development level. The following conclusions can be drawn:

(1) The industrial green manufacturing development level within the Yangtze River Economic Zone has exhibited a generally upward trajectory, characterized by an average annual growth rate of 5%. The degree of industrial green manufacturing development varies greatly throughout the region’s areas, exhibiting a “strong in the east and weak in the west” pattern of development. The development level of industrial green manufacturing can be categorized into five levels: low, low, medium, high, and high. Notably, the high tiers are predominantly found in central and eastern regions, including Jiangsu, Zhejiang, Shanghai, and Anhui, whereas the low tier is more prevalent in the western regions, such as Yunnan. There are notable regional variations, with a decreasing development level from the east to the west of the region’s spatial distribution pattern. However, in terms of the trend of dynamic change, the region with a high level of industrial green manufacturing development as well as a higher level gradually expands and increases from east to west as time progresses.
(2) From 2018 to 2022, the spatial disparities in the growth of industrial green manufacturing inside the Yangtze River Economic Belt grew, and by 2022, they narrowed. Due to initial conditions, there is a certain “Matthew effect” and inter-regional differences account for the majority of the overall differences between the three regions. The eastern region of the Yangtze River Economic Belt is at the forefront of industrial green manufacturing development. The trajectory of intra-regional disparities and their contribution rates exhibits an overall pattern of fluctuation and escalation, with the central region displaying the most significant intra-regional disparities and contribution rates, succeeded by the eastern region. In contrast, the western region records comparatively lower rates, and the issue of intra-regional disparities is gaining increasing prominence.
(3) The industrial green development within the Yangtze River Economic Belt demonstrates a pronounced positive spatial correlation, indicative of a spatial spillover effect. The development levels of green manufacturing in adjacent regions are interdependent, yet there is a noted deceleration in the trend of spatial agglomeration and distribution. The Moran scatter plot shows that the majority of provinces and cities fall into either the HH-type or the LL-type. The HH-type is primarily found in eastern provinces and cities like Zhejiang and Jiangsu, while the LL-type is more prevalent in central and western provinces and cities. The data indicates a pronounced east-west disparity in China’s industrial green manufacturing development, characterized by a “strong in the east, weak in the west” distribution pattern. However, the number of HH-type provinces has been increasing somewhat over time.

Based on the study, the future should adhere to the drive of science and technology innovation, especially in clean energy, energy saving and emission reduction, and circular economy, while coordinating and balancing the promotion of green and sustainable development, expanding and opening up to help improve the tenacity of the development of green manufacturing. To elevate the level of industrial green manufacturing across the Yangtze River Economic Belt by addressing regional disparities in development levels and fostering high-quality, coordinated regional development in the sector [60]. Furthermore, the central and western regions within the Yangtze River Economic Belt face limitations due to their economic development levels and additional factors, resulting in significant disparities when compared to the economically advanced cities along the eastern seaboard. Henceforth, it is imperative to augment investment in infrastructural development within the central and western regions of the Yangtze River Economic Belt, thereby enhancing transportation, energy, and information infrastructure. This strategy aims to bolster the region’s appeal and establish a conducive physical environment for the advancement of industrial green manufacturing. To foster balanced regional economic development, this study advocates for enhanced inter-regional industrial collaboration and the strategic relocation of industries from the eastern region to the central and western regions. Concurrently, it is essential to direct the migration of capital, technology, and human resources towards these areas, thereby facilitating equilibrium in regional economic growth. Furthermore, enhancing spatial agglomeration can be achieved by leveraging the spatial spillover effect, thereby accelerating the advancement of industrial green manufacturing development.

## Supporting information

S1 File This file contains minimal data. (XLSX)
